# Nanocomposite Films of Silver Nanoparticles and Conjugated Copolymer in Natural and Nano-Form: Structural and Morphological Studies

**DOI:** 10.3390/ma16103663

**Published:** 2023-05-11

**Authors:** Rebeca da Rocha Rodrigues, Diogo Silva Pellosi, Guy Louarn, Laura Oliveira Péres

**Affiliations:** 1 Department of Exact and Earth Sciences, Federal University of São Paulo, UNIFESP, Campus Diadema, São Paulo 09913-030, Brazil; rebeca.rodrigues@unifesp.br (R.d.R.R.); diogo.pellosi@unifesp.br (D.S.P.); laura.peres@unifesp.br (L.O.P.); 2 Institut des Matériaux de Nantes Jean Rouxel, IMN, Nantes Université, CNRS, F-44000 Nantes, France

**Keywords:** PEDOT, polyfluorene, plasmonic nanoparticles, Raman spectroscopy, sensors

## Abstract

The use of conjugated polymers (CPs) and metallic nanoparticles is an interesting way to form nanocomposites with improved optical properties. For instance, a nanocomposite with high sensitivity can be produced. However, the hydrophobicity of CPs may hamper applications due to their low bioavailability and low operability in aqueous media. This problem can be overcome by forming thin solid films from an aqueous dispersion containing small CP nanoparticles. So, in this work we developed the formation of thin films of poly(9,9-dioctylfluorene-co-3,4-ethylenedioxythiophene) (PDOF-co-PEDOT) from its natural and nano form (NCP) from aqueous solution. These copolymers were then blended in films with triangular and spherical silver nanoparticles (AgNP) for future applicability as a SERS sensor of pesticides. TEM characterization showed that the AgNP were adsorbed on the NCP surface, forming a nanostructure with an average diameter of 90 nm (value according to that obtained by DLS) and with a negative potential zeta. These nanostructures were transferred to a solid substrate, forming thin and homogeneous films with different morphology of PDOF-co-PEDOT films, as observed by atomic force microscopy (AFM). XPS data demonstrated the presence of the AgNP in the thin films, as well as evidence that films with NCP are more resistant to the photo-oxidation process. Raman spectra showed characteristic peaks of the copolymer in the films prepared with NCP. It should also be noted the enhancement effect of Raman bands observed on films containing AgNP, a strong indication of the SERS effect induced by the metallic nanoparticles. Furthermore, the different geometry of the AgNP influences the way in which the adsorption between the NCP and the metal surface occurs, with a perpendicular adsorption between the NCP chains and the surface of the triangular AgNP.

## 1. Introduction

Since the discovery of conjugated polymers (CPs), the use of these materials in different applications has been intensively evaluated, such as in light-emitting diodes (LED), solar cells, and sensors [1]. Due to its favorable electrical, thermal, and optical properties, the use of CPs in sensor development provides several advantages such as structural stability, improvement of response time, sensibility, and specificity of the sensor [1,2]. In particular, polyfluorene (PF) and poly(3,4-ethylenedioxythiophene) (PEDOT) show promising properties for application as sensors, such as a high emission in the blue region for PF [1], in addition to the high conductivity, biocompatibility, and feasibility to functionalization of PEDOT [3].

A factor to be highlighted about CPs is the relationship between the architecture of polymeric systems and their intrinsic properties. Thus, the preparation of polymeric films with an ordering degree can provide an improvement in the properties of CPs [1]. Among the most used techniques for the formation of these films are Spin Coating [4,5] and Drop-Casting [5,6], which are simple techniques based on the coating of solid supports by centrifugal force or solvent evaporation, respectively.

The improvement of the properties of CPs can also be achieved with the insertion of electron-rich materials, such as metallic nanoparticles, resulting in the formation of nanocomposites with improved thermal, optical, mechanical, and conductive properties [7,8,9]. Compared to the metal on a macroscopic scale, metallic nanoparticles have unique physical, chemical, biological, electric, and photophysical properties, being used in several applications such as photocatalysis [10], medicine [11], electronic and optical devices [12]. Among the metals, silver is extremely used for the formation of nanoparticles for various applications, due to its high thermal and electrical conductivity, its antibacterial properties and modulation of its optical properties depending on the shape and size of the nanoparticle [8,9,13]. Silver nanoparticles also have the ability to promote the surface-enhanced Raman scattering effect (SERS), which significantly increases the sensitivity for detecting an analyte, allowing the formation of so-called SERS sensors [8,14].

However, the hydrophobicity of CPs can make its applications difficult due to its low bioavailability and operability in aqueous media [15]. Among the strategies that can be used, the preparation of a polymeric dispersion in water or buffer solution can be carried out, resulting in CPs nanoparticles [15,16]. With these ideas in mind, we aimed to prepare and analyse the properties of thin films based on nanocomposites of the unpublished copolymer poly(9,9-dioctylfluorene-*co*-3,4-ethylenedioxythiophene) (PDOF-co-PEDOT) in its natural and nanoparticle form (NCP), blended with silver nanoparticles (AgNP) of two different shapes (spherical and triangular) deposited onto a glass or quartz slide, aiming to understand the morphological and structural properties of these unpublished nanocomposites for future application as SERS sensors.

## 2. Materials and Methods

### 2.1. Synthesis of the Copolymer and Silver Nanoparticles

The copolymer poly(9,9-dioctylfluorene-*co*-3,4-ethylenedioxythiophene) (PDOF-co-PEDOT) was synthesized according to the Suzuki route [17,18] and the complete synthesis methodology is described in Supporting Information. The brown solid obtained was characterized by Fourier transform infrared spectroscopy (FTIR), UV-vis spectroscopy (UV-vis), fluorescence emission, X-ray photon-electron spectroscopy (XPS), thermal analysis (TGA) and differential scanning calorimetry (DSC).

Spherical and triangular silver nanoparticles (AgNP) were synthesized based on the methodology described by Silva et al. [19]. The synthesis is described in Supporting Information and it should be mentioned that the glassware to be used in the synthesis was previously cleaned with a 2 M HNO_3_ solution and then rinsed thoroughly with ultrapure water. The AgNP dispersions were characterized by UV-vis spectroscopy, dynamic light scattering (DLS), zeta potential, transmission electron microscopy (TEM), and energy dispersive spectroscopy (EDS).

### 2.2. Preparation and Characterization of Nanocopolymer (NCP)

The NCP was prepared by solvent exchange/nanoprecipitation method [16,20]. In this methodology, a solution of the polymer in a good and water-miscible solvent was added to water. Due to this, the solubility of the polymer drastically decreases, curling on a nanometer scale and precipitating. In a beaker, 3 mL of PDOF-co-PEDOT solution (0.5 mg/L in THF) were added to 12 mL of Milli-Q water. The solution was left under stirring until total evaporation of THF solvent and the dispersion obtained was characterized by UV-vis, TEM, DLS, and zeta potential.

### 2.3. Preparation and Characterization of Spin Coating and Casting Films

In the case of NCP/AgNP films, consecutive layers of an aqueous dispersion of NCP and AgNP (ratio of NCP/AgNP = 1:7 *v*/*v*) were deposited using the Spin Coating technique (250 µL, 400 rpm, time during maximum rotation of 40 s) in glass or quartz substrate (for the UV-vis analysis). For better deposition, the NCP/AgNP layers were alternating with layers of 3-aminopropyltriethoxysilane, with the immersion of the films in a 3% solution of the aminosilane in anhydrous toluene for 30 min.

For the preparation of the films containing PDOF-co-PEDOT/AgNP, alternating layers were deposited by Spin Coating (for the PDOF-co-PEDOT) and Casting techniques (for the AgNP). For the copolymer layers, a solution of 0.5 mg/L in chloroform of PDOF-co-PEDOT was used, with Spin parameters of 250 µL, 400 rpm and time during maximum rotation of 40 s. The AgNP layers were deposited by Casting technique using 250 µL of the AgNP dispersion. Prior to deposition of alternate layers, a 3-aminopropyltriethoxysilane was deposited on the substrate. During the preparation of all films, the layers were dried at 50 °C. The films were characterized by UV-vis, atomic force microscopy (AFM), XPS and Raman spectroscopy.

### 2.4. Characterization Techniques

For the PDOF-co-PEDOT synthesized the FTIR spectra were obtained by a Shimadzu spectrophotometer, model IR-Prestige 21. The analyses were performed with 256 scans, 4 cm^−1^ of resolution, using diffuse reflectance accessory (DRIFT) and potassium bromide (KBr) as background. A Horiba fluorimeter (Fluorog FL3C-22) was used for the fluorescence emission analysis, using a quartz cuvette of 1 cm, integration time of 0.1 s, and excitation and emission slits of 3.0 mm. TGA analyses were performed by thermal analyser Discovery SDT 650 (TA Instruments—New Castle, DE, USA) in a range of 30 to 800 °C with a heating rate of 10 °C·min^−1^, nitrogen flow of 50 mL·min^−1^ and approximated 5 mg of the sample.

The absorption spectra of PDOF-co-PEDOT, NCP and AgNP solutions were obtained by Cary-60 spectrophotometer (Agilent Technologies—Santa Clara, CA, USA) using a wavelength of 200 to 1100 nm, quartz cuvette of 1 cm and integration time of 0.1 s. A special support for films was used in UV-vis spectroscopy. TEM images and EDS analysis of the NCP and AgNP dispersion were obtained by JEOL JEM 2100 equipment coupled to an energy-dispersed detector. The samples were deposited in 300 Mesh copper grids with carbon support (Lacey Carbon grid), 50 nm. DLS and zeta potential analyses were performed with a Zetasizer Nano ZS (Malvern Instruments—Malvern, UK) using a He-Ne laser of 4 mV in a wavelength of 633 nm. The dispersions were added in capillary cuvette DTS 1070 without previous sample treatment. The analyses were carried out at a constant temperature of 20 °C with a refraction index of 0.14.

AFM images of the films were performed in tapping mode on a Multimode 8 Nanoscope V (Bruker—Paris, FR) using a silicon tip on nitride lever (Bruker, model Scanasyst-air) with an imaging resonance frequency of 70 kHz and an elastic coefficient of 0.4 N/m. The images obtained were analysed with Gwyddion software (version 2.62). The XPS analyses were carried out at room temperature on Axis Nova spectrometer (Kratos Analytical—Manchester, UK) using the Al Kα line (1486.6 eV) as an excitation source. Data analyses were performed using the software CasaXPS (version 2.1.0.1). For calibration, the binding energy for the C_1s_ peak was set at 284.8 eV, with a constant pass energy of 20 eV and resolution of 0.22 eV. Raman spectra of the films were obtained by a Renishaw InVia reflex spectrometer at room temperature and in air atmosphere (λ_exc_ = 514 nm, laser power = 10 mW and exposure time 20 s).

## 3. Results and Discussion

### 3.1. Characterizations of Pristine Materials and Dispersions of NCP and AgNP

The structural characteristics and thermal properties of the synthesized PDOF-co-PEDOT were studied by FTIR and TGA techniques and shown in Figure S1, in the supporting information. For the FTIR spectra (Figure S1A,B), bands at 2955, 2926 and 2853 cm^−1^ correspond to C-H stretches associated to saturated hydrogens present in the polymeric chain. The C=C stretches of fluorene rings are indicated due to the presence of the bands at 1585 and 1510 cm^−1^ bands. In turn, the bands at 1360 and 1088 cm^−1^ are attributed to the C-S-C stretch and asymmetric C-O-C stretch of the EDOT group, respectively. The bands at 890–810 cm^−1^ are attributed to the symmetric C-O-C stretch of the EDOT group and the out-of-plane C-H angular deformation of the fluorene rings [18,21,22]. Therefore, FTIR analysis confirms co-polymer structure formation.

From the DSC curve of PDOF-co-PEDOT, represented in Figure S1D, an exothermic peak can be observed at 238.1 °C. TGA and DTG curves (Figure S1C), showed two weight loss events in the degradation of the copolymer. The first event corresponds to a loss of 9.4% of initial mass with an onset temperature of 232.98 °C, and a second more pronounced event with degradation of 54.8% of the PDOF-co-PEDOT with onset temperature of 405.1 °C. In the literature, analogous copolymers containing fluorene and EDOT groups showed thermal degradation events with onset temperatures in a region between 350–450 °C [21,22]. As reported [22], the presence of the cyclic ether in PEDOT causes a lower thermal stability of the copolymer. Therefore, the first and second thermal events correspond, respectively, to PEDOT and polyfluorene degradation.

The optoelectronic properties were studied by absorption in UV-vis and fluorescence. Figure S2A,B shows the absorption and emission spectra of PDOF-co-PEDOT, which has maximum absorptions at 383 and 430 nm (2.61 eV) and three emission peaks at 415, 456 and 484 nm. These values following those reported in the literature for analogous copolymers of fluorene and thiophene [21,22,23]. Analysing fluorescence emission of the NCP, there was a broadening and red-shift of the maximum absorption and emission, in addition to the loss of definition of the emission band compared to PDOF-co-PEDOT (Figure S2). It can be assumed that in the nano format, the copolymer may be forming a J-type aggregate in which the dipoles align side by side, resulting in the observed bathochromic shift [24,25].

AgNPs were characterized by UV-vis absorption (Figure S2A). Spherical nanoparticles are characterized by a plasmonic band at approximately 400 nm [13]. Differently, triangular AgNPs showed a broad band with maximum at 730 nm and a less intense band at 330 nm. Anisotropic nanoparticles, such as triangular AgNP, are characterized by a band influenced by two factors: (i) the dipole resonance, being similar to spherical nanoparticles, having a less intense band in a region close to 400 nm; and (ii) quadrupole resonance, corresponding to the plasmonic band with higher intensity, extremely sensitive to the size of the nanoparticle and at wavelengths higher than 500 nm [26]. Dispersions containing NCP and AgNP were prepared and their absorption and emission spectra are show in Figure S2A,B. For both systems, absorption spectra showed the characteristic bands of NCP and AgNP (with only small shifts of the absorption maxima), while the emission bands showed a similar profile to the NCP emission band. These studies involving the optoelectronic properties of the composite were important to determining the best *v*/*v* ratio between the NCP and AgNP. The proportion chosen was the one that presented an absorption and emission spectrum in which both components present their respective signals, in addition to a higher concentration of AgNP, an important component for future applications, as SERS sensors.

Based on DLS and zeta potential data (Table 1) and particle size and zeta distribution images showed in Supporting Information (Figures S3 and S4), NCP showed a larger size when compared to AgNP, in addition to a neutral potential. It should be noted that the negative potential of AgNP is due to the protective effect of citrate ions around the AgNP [27]. In addition, for both NCP and AgNP, the negative zeta potential values indicate the stability of the dispersions (values between ±30 mV).

**Figure 1:**
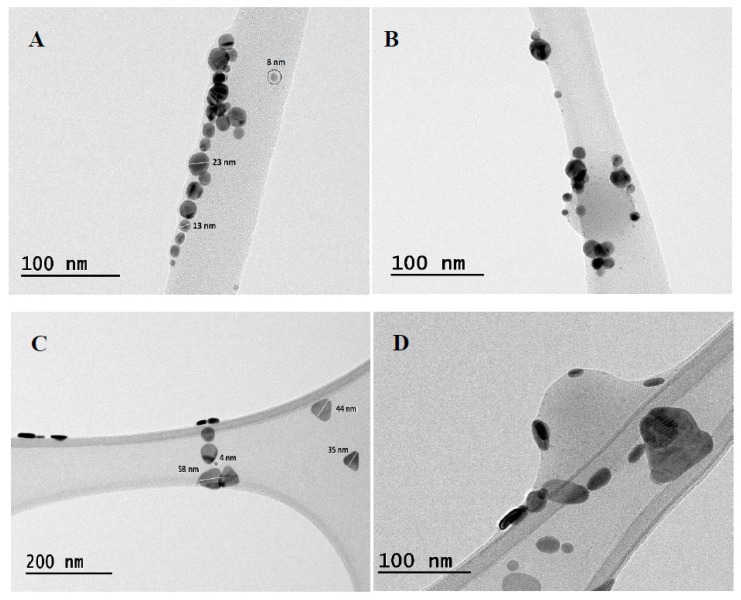
TEM images of the spherical (**A**) and triangular AgNP (**C**) and the nanocomposites of NCP with spherical (**B**) and triangular AgNP (**D**).

TEM micrographs and EDS analyses of the pristine materials (AgNP and NCP) and NCP/AgNP dispersions were performed, as shown in Figure 1, Figures S5 and S6 in the supporting information. From these data, it was possible to verify the composition, size and geometry of the synthesized AgNP (Figure 1A,C and Figure S6A) and NCP (Figures S5 and S6B). Figure 1A,C demonstrate the spherical and triangular shape of the synthesized AgNP. The triangular nanoparticle presented oval corners that can also be noted when these nanoparticles are edge-oriented [28], or observed when this AgNP is in contact with the NCP (Figure 1D). For both spherical and triangular AgNP, nanoparticles with sizes close to the values obtained by DLS are observed. In addition, there are nanoparticles of smaller sizes (4–8 nm), called seeds nanoparticles, which are formed in the first step of nucleation and growth mechanism responsible for the formation of the nanoparticles [29]. The NCP presented a spherical shape and diameter value close to the obtained in the DLS analyses. Something interesting to note for the dispersion’s analyses (Figure 1B,D) is the formation of structures in which AgNPs are adsorbed on the NCP surface, suggesting that the mechanism formation for NCP/AgNP is the adsorption of the silver nanostructures onto the nanopolymer surface by specific interactions of Ag with copolymer sulphur molecules.

### 3.2. Films Containing PDOF-co-PEDOT or NCP with AgNP

**Figure 2:**
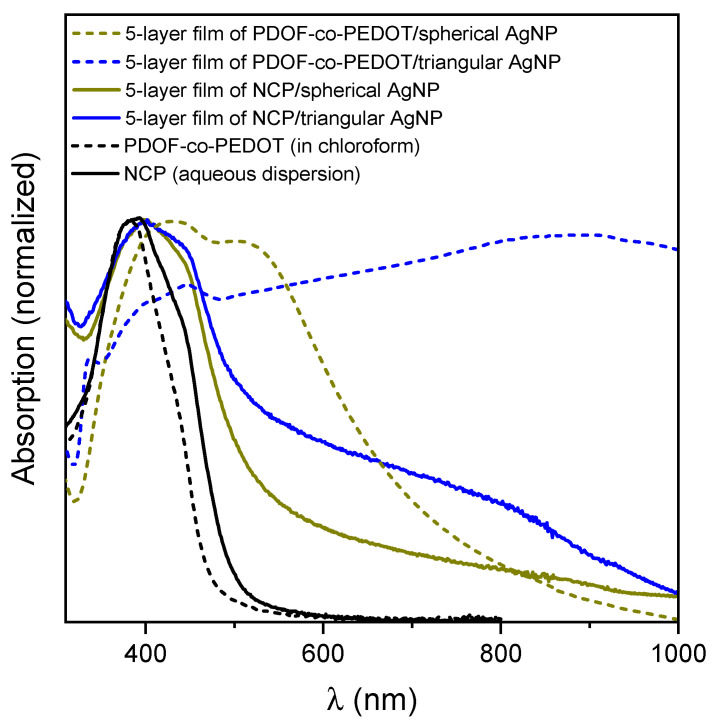
UV-vis spectra of 5-layer film of PDOF-co-PEDOT or NCP with AgNP, and spectra of PDOF-co-PEDOT and NCP in chloroform and water, respectively.

In glass or quartz substrate, five-layer films of AgNPs with PDOF-co-PEDOT or NCP were prepared and characterized. Figure 2 shows the UV-vis spectra of the supported films (in quartz) and, for comparison, PDOF-co-PEDOT and NCP solutions spectra. For the films, there was a bathochromic shift and a broadening of the absorption bands, in addition to the appearance of the SPR band of triangular AgNP for the films containing this nanoparticle. Clearly, these shifts and broadening of PDOF-co-PEDOT and NCP bands are due to the AgNP adsorption. However, solid-state polymeric systems also are susceptible to excimer formation. With this in mind, it is possible to observe in the emission spectrum of PDOF-co-PEDOT film (Figure S7) a less defined emission bands shifted to higher wavelengths, characterizing the formation of these complexes [30,31].

**Figure 3:**
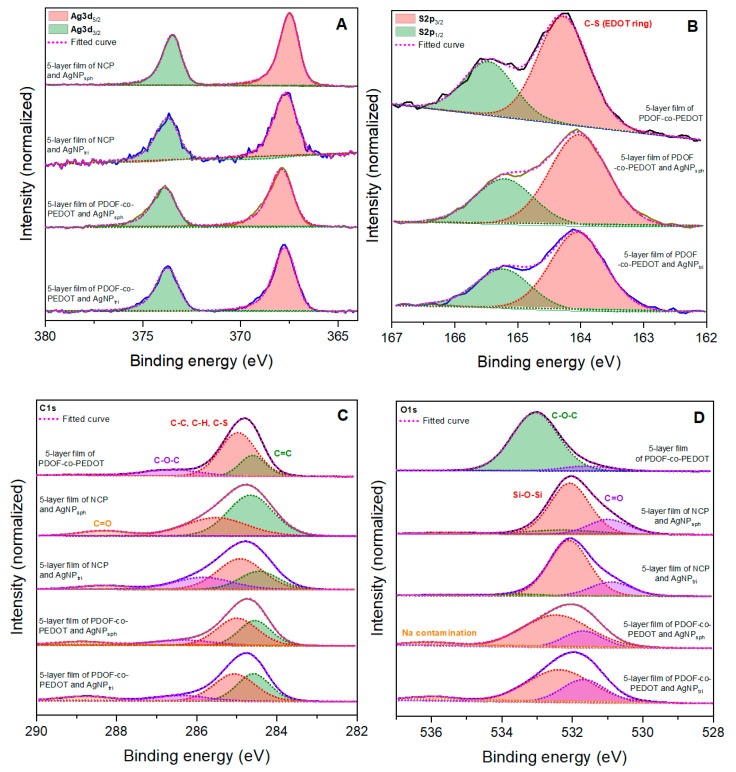
Ag3d (**A**), S2p (**B**), C1s (**C**) and O1s (**D**) XPS spectra of neat 5-layer film of PDOF-co-PEDOT and 5-layer films of NCP and PDOF-co-PEDOT with spherical and triangular AgNP.

To analyse the chemical composition of the films, XPS experiments were carried out, as shown in Figure S8 and Figure 3A–D. For all films with AgNP the characteristic peaks of Ag3d 5/2 and Ag3d 3/2 appeared at 367.6 ± 0.2 eV and 373.6 ± 0.2 eV (spin-orbit splitting = 6.0 eV), respectively (Figure 3A). These values are in accordance to the reported values for zero-valent silver [32,33], confirming the metallic zero-valent state for Ag in the silver nanoparticle structure. In S2p XPS spectra (Figure 3B) the films with PDOF-co-PEDOT showed the presence of two peaks at 164.1 ± 0.1 eV and 165.3 ± 0.1 eV that corresponds to the C-S bond of the EDOT rings in the copolymer [34,35].

The C1s XPS spectrum (Figure 3C) of the neat five-layer film of PDOF-co-PEDOT was decomposed in three peaks at 284.6, 284.9 and 286.6 eV, that can be attributed to C=C, C-C/C-H/C-S and C-O-C bonds, respectively [36,37,38]. Unlike the neat film, in the C1s XPS spectra of the films with AgNP a different peak appeared at 288.6 ± 0.3 eV. This peak characterizes the presence of carbonyl and carboxyl groups from photo-oxidative degradation [36,38], and this degradation is due to close contact between organic molecules and SERS-active metal nanoparticles [39]. The O1s XPS spectrum (Figure 3D) of the neat film presents two peaks at 533.0 and 531.6 eV correspond to C-O-C and C=O bonds [36,40]. On the other hand, for the films with AgNP, the peaks at 532.3 ± 0.1 eV and 531.5 ± 0.4 eV correspond to Si-O-Si (substrate or 3-aminopropyltriethoxysilane) and C=O (carbonyl/carboxyl groups) [41]. Additionally, the peak at 535.7 ± 0.3 eV (Figure 3D) that appears in the spectra of films with AgNP (in a higher percentage for the PDOF-co-PEDOT/AgNP films) is related to sodium contamination [36] probably due sodium borohydride from AgNP synthesis. In Figure S8 we could also note peaks of nitrogen, corresponding to the C-N bond of 3-aminopropyltriethoxysilane, used for better film deposition.

Quantitative XPS studies based on peak areas of interest were performed (Table 2). Analysing the relative percentage (area) corresponding to carbonyl/carboxyl groups in the O1s spectra for films with AgNP, it is noteworthy that the films composed by PDOF-co-PEDOT in its natural form with any AgNP showed a higher percentage of carbonyl/carboxyl groups. The same results can be observed for the C=O peak of C1s spectra. This is interesting, because perhaps the films composed of the PDOF-co-PEDOT in its nano form (NCP) may be more resistant to photodegradation, which would be an interesting study in the future.

**Figure 4:**
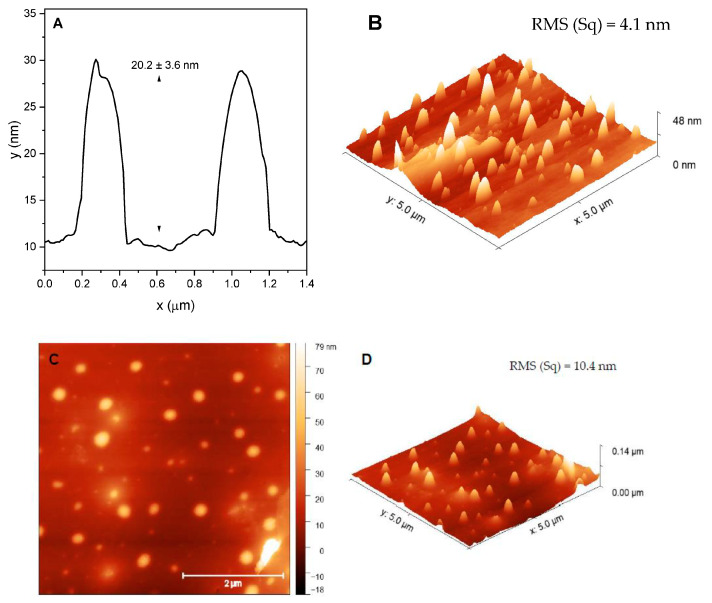
(**A**) profile along the y-axis of the 5-layer film of NCP/spherical AgNP and its 3D image (**B**); and AFM (5 × 5 µm) and 3D image (**C**,**D**, respectively) of 5-layer film of NCP/triangular AgNP.

The evaluation of the film’s morphology was performed by AFM, and some images and results are shown in Figure 4 and Figure S9. Comparing the AFM and 3D images, the films containing NCP (Figure 4B–D) show a very different morphology from films with PDOF-co-PEDOT in its natural form (Figure S9A,B). NCP films with AgNP showed circular structures scattered on their surface with an average size of 20.2 ± 3.6 nm (Figure 4A) and 32.7 ± 5.7 nm for films with AgNP spherical and triangular, respectively. These structures were probably formed due to the interaction between NCP and AgNP, which has already been identified in previous analyses (zeta potential, DLS and TEM). It is also highlighted that regardless of the copolymer form used in the films, those with triangular AgNPs showed higher RMS values, being 10.4 and 5.4 nm for NCP/triangular AgNP and PDOF-co-PEDOT/triangular AgNP films, respectively. This demonstrates the influence of the AgNP geometry on the morphology of the films, which in turn may influence the obtained SERS spectra obtained from copolymer AgNP films as discussed below.

**Figure 5:**
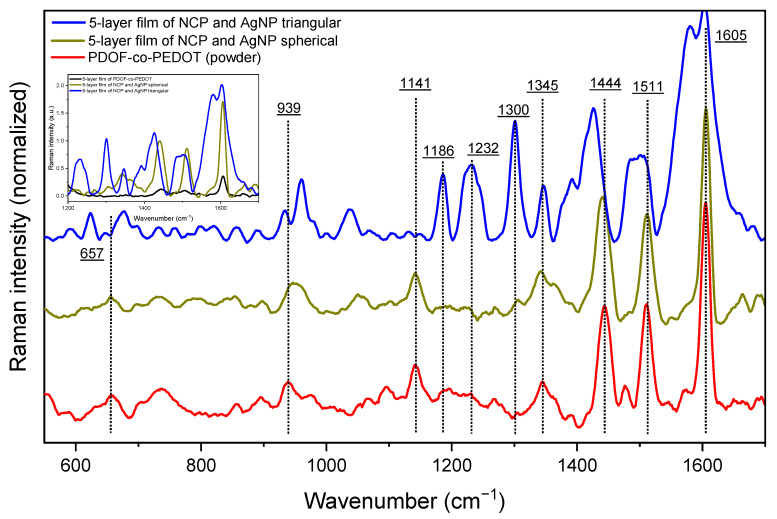
Raman spectra of 5-layer films and copolymer (powder) wit normalized Raman intensity and inset graph with Raman spectra between 1000–1800 cm^−1^.

Raman spectroscopy is an interesting technique that allows the obtaining characteristic spectra of molecular structures. Thus, any change/shift of the spectral bands could be a result of different interactions between the polymer and the AgNP. Figure 5 compares the spectra of the films with NCP and AgNP with the spectra of the powder and film (inset) copolymer. For the PDOF-co-PEDOT pure (powder), the most intense band is located at 1605 cm^−1^, being assigned to the phenyl intra-ring C-C stretch mode of the fluorene group [6,42,43]. The other less intense bands are listed in Table 3 with their respective attributions. It is worth noting that lower intensity bands between 1350–1250 cm^−1^, 1250–1170 cm^−1^, and 1170–1100 cm^−1^ are characteristic bands of interphenyl ring backbone C-C stretch mode, and C-H bending modes from backbone and side chains of fluorene groups, respectively [42].

When the AgNP are added to form the films, an effect of Raman band enhancement (Figure 5—inset) can be observed, a strong indication of the SERS effect induced by metallic nanoparticles and a promising factor for the use of these nanocomposite films as sensors.

Some spectral differences can also be observed for the films:

(i) band shifts, mainly for the film with triangular AgNP;
(ii) increase in the relative intensities of the bands between 1350–1180 cm^−1^ and the band at 939 cm^−1^ for the film with triangular AgNP.

The highlighted spectral differences demonstrate the interaction between AgNP and NCP and that this interaction does not occur in the same way for the spherical and triangular AgNP. Regarding the increase in the relative intensity of certain bands, the SERS selection rules state that the modes perpendicular to the metal surface are more intense than the parallel modes [44,46]. As these more intense bands are characteristic of the oxyethylene ring deformation of PEDOT and interphenyl ring backbone C-C stretch mode and C-H bending modes from backbone of the fluorene rings, NCP adsorption on films containing triangular AgNP would be perpendicular to the surface of the silver nanoparticle.

## 4. Conclusions

The preparation of films composed of nanohybrid systems between conjugated polymers and different-shaped silver nanoparticles was successfully performed and characterized. The junction of the two materials alters, as expected, morphological and electronic properties of both materials as demonstrated by UV-Vis, fluorescence, and TEM images. Dispersions composed of NCP and AgNP demonstrated the formation of interesting structures that could be transferred to a solid support, resulting in a different morphology from that obtained for films containing the copolymer in natural form. XPS and AFM characterizations demonstrated that the different geometry of the AgNP also influences the morphology of all films, since films with triangular AgNP presented higher RMS values. In addition, the SERS effect of the films with NCP has been proven by initial Raman results, since there was an intensification of the characteristic bands of the copolymer in the films. The different geometries of the silver nanoparticles also influence how the adsorption between the NCP and the metallic surface occurs.

Finally, the authors hope that this work may contribute to the scientific community in clarifying how systems composed of a copolymer of polyfluorene and thiophene and metallic nanoparticles behave in face of the different size/dimension of the copolymer and the different geometry of the metallic nanoparticles. With the properties discussed in this work and with ongoing studies, the authors hope that these specific films can be applied as sensors in the future, even in applications where photo-oxidation processes are something important to be considered.

## Figures and Tables

**Table 1:** Hydrodynamic diameter, polydispersity (PDI) and zeta potential average values of AgNP, NCP and dispersions with NCP and AgNP.

	Diameter (nm)	PDI	Zeta Potential (mV)
Spherical AgNP	13.37	0.61	−29.50
Triangular AgNP	28.65	0.61	−37.80
Nanocopolymer (NCP)	99.96	0.11	0.02
NCP/spherical AgNP	93.57	0.14	−49.60
NCP/triangular AgNP	82.09	0.65	−43.00

**Table 2:** Relative percentages of the characteristic peaks of C=O and Na contamination for O1s and C1s XPS spectra for the 5-layer films with AgNP.

5-Layer Film	O1s (% Area)		C1s (% Area)
	C=O	Na Contamination	C=O
NCP/spherical AgNP	20.04	2.38	7.62
NCP/triangular AgNP	19.28	1.86	6.25
PDOF-co-PEDOT/spherical AgNP	22.15	5.51	8.35
PDOF-co-PEDOT/triangular AgNP	30.41	6.48	10.04

**Table 3:** The main observed Raman frequencies (cm^−1^) of powder PDOF-co-PEDOT with their respective assignments.

Raman Signal (cm^−1^)	Assignments	Literature
1511	asymmetric Cα=Cβ stretch mode of the thiophene ring	[39]
1444	symmetric Cα=Cβ stretch mode of thiophene	[44]
1345	Cβ-Cβ stretch mode of inter-ring bonds of thiophene	[44,45]
1141	C-H bending modes from side chains of fluorene groups	[42]
939	oxyethylene ring deformation	[44]
657	C-S-C stretch mode	[45]

## Data Availability

Not applicable.

## Supplementary Materials

The following supporting information can be downloaded at: https://www.mdpi.com/article/10.3390/ma16103663/s1, Figure S1. FTIR spectra in the range of 3200 to 2600 cm^−1^ (A) and 1650 to 400 cm^−1^ (B); TGA with its derivative curve (C) and DSC of PDOF-co-PEDOT (D); Figure S2. (A) Absorption spectra of the pristine PDOF-co-PEDOT (chloroform—6 mg/L), NCP (aqueous dispersion—60 mg/L), spherical and triangular AgNP (aqueous dispersion—60 mg/L), and dispersions of NCP/AgNP (60 mg/L); and (B) Emission spectra with normalized intensity of PDOF-co-PEDOT, NCP and dispersions of NCP/AgNP; Figure S3. Particle size distribution of spherical (A) and triangular (B) AgNP, NCP/spherical AgNP (C), NCP/triangular AgNP (D) and NCP (E); Figure S4. Zeta distribution of spherical (A) and triangular (B) AgNP, NCP/spherical AgNP (C), NCP/triangular AgNP (D) and NCP (E); Figure S5. TEM images of NCP; Figure S6. EDS spectra of AgNP (A) and NCP (B); Figure S7. Emission spectra of PDOF-co-PEDOT in solution (black curve) and solid-state (red curve); Figure S8. XPS spectra of five-layer films of PDOF-co-PEDOT and five-layer films of NCP or PDOF-co-PEDOT with spherical and triangular AgNP; Figure S9. AFM images and their respective peak force error and 3D images of five-layer films of PDOF-co-PEDOT with spherical (A) and triangular (B) AgNP.
