# Supplementary material for: Nanocomposite Films of Silver Nanoparticles and Conjugated Copolymer in Natural and Nano-Form: Structural and Morphological Studies
Source: Materials (Basel). 2023 May 11;16(10):3663. doi: 10.3390/ma16103663 (PMC10223115; doi:10.3390/ma16103663)
Supplement: Supplementary file 1 [file materials-16-03663-s001.zip › materials-2352375-supplementary.pdf]

Article

Nanocomposite films of silver nanoparticles and conjugated copolymer in natural and nano-form: structural and morphological studies

Rebeca da Rocha Rodrigues [1,2], Diogo Silva Pellosi [1], Guy Louarn [2,*] and Laura Oliveira Péres [1]

[1] Federal University of São Paulo, UNIFESP, Department of Exact and Earth Sciences, Campus Diadema, 09913-030 São Paulo, Brazil; rebeca.rodrigues@unifesp.br (R.d.R.R.); diogo.pellosi@unifesp.br (D.S.P.); laura.peres@unifesp.br (L.O.P.)

[2] Nantes Université, CNRS, Institut des Matériaux de Nantes Jean Rouxel, IMN, F-44000 Nantes, France

* Correspondence: guy.louarn@cnrs-imn.fr

Supporting Information

Materials and Methods section:

- **Synthesis of the copolymer PDOF-co-PEDOT**

Under reflux system and inert atmosphere (N_2) in a three-neck flask, the monomers 2,5-dibromo-3,4-ethylenedioxythiophene (3.55 mmol) and 9,9-dioctyl-fluorene-2,7-diboronic acid bis(pinacol) ester (3.05 mmol) were added with toluene and the catalyst tetrakis(triphenylphosphine)palladium (0) (0.01 mmol). After 72 hours, was added 9,9-dioctyl-fluorene- 2-boronic acid pinacol ester (0.5 mmol) and the synthesis is left under the same conditions as above for 24 hours. The copolymer was extracted and purified by Soxhlet extraction, using methanol as solvent.

- **Synthesis of the spherical and triangular silver nanoparticles (AgNP)**

For spherical AgNP, 20 mL of mili-Q water, 2.5 mL of sodium citrate (0.017 mol/L), 2.5 mL of silver nitrate (0.001 mol/L) and 250 μL of sodium borohydride (0.1 mol/L) were added in an erlenmeyer and left under magnetic stirring at room temperature for 1 hour. In the case of triangular AgNP the same methodology was used, but before the addition of sodium borohydride were added 60 μL of hydrogen peroxide (Synth – 29% v/v).

Results and discussion section:

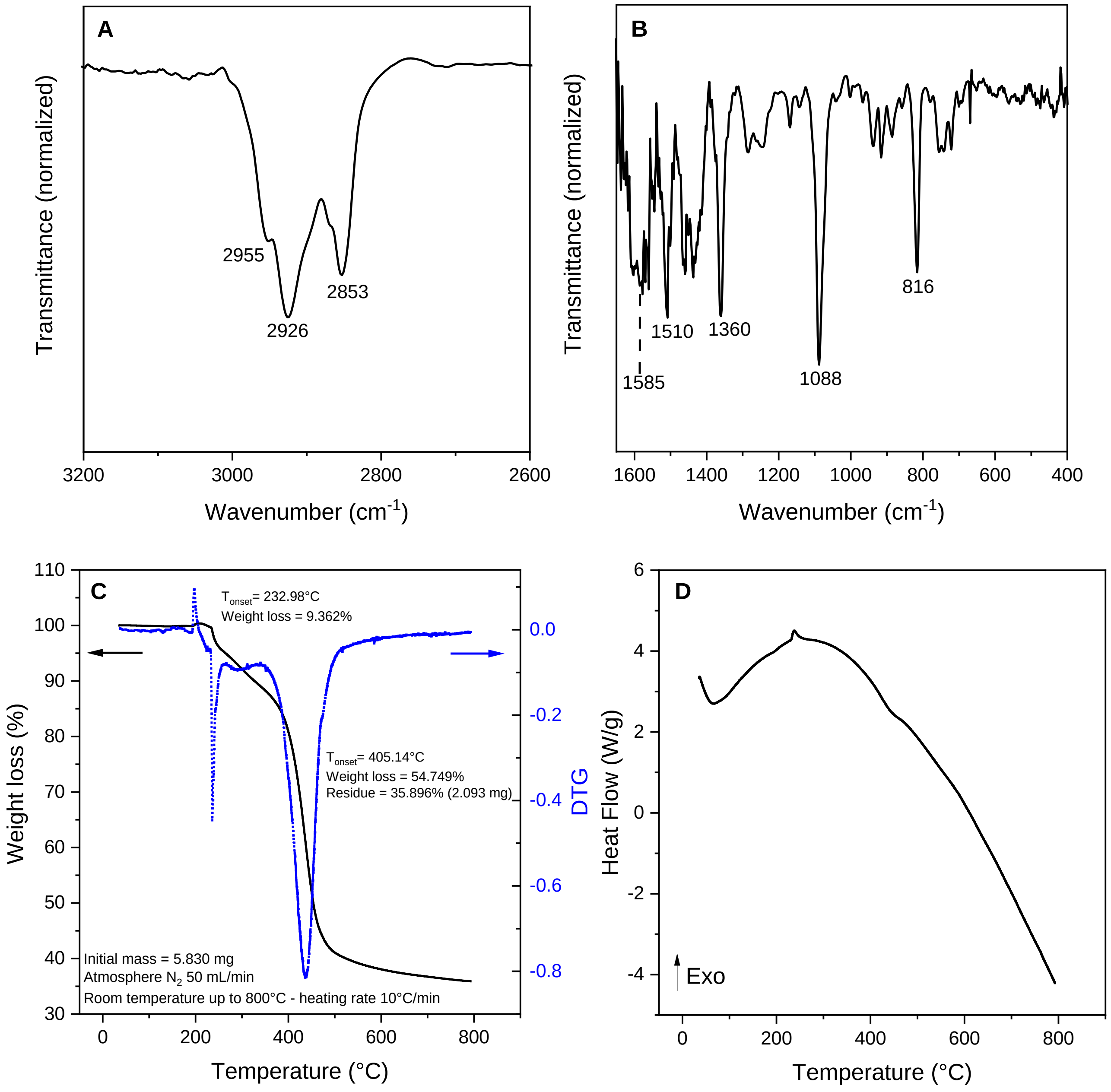

Figure S1. FTIR spectra in the range of 3200 to 2600 cm^{-1} (**A**) and 1650 to 400 cm^{-1} (**B**); TGA with its derivative curve (**C**) and DSC of PDOF-co-PEDOT (**D**).

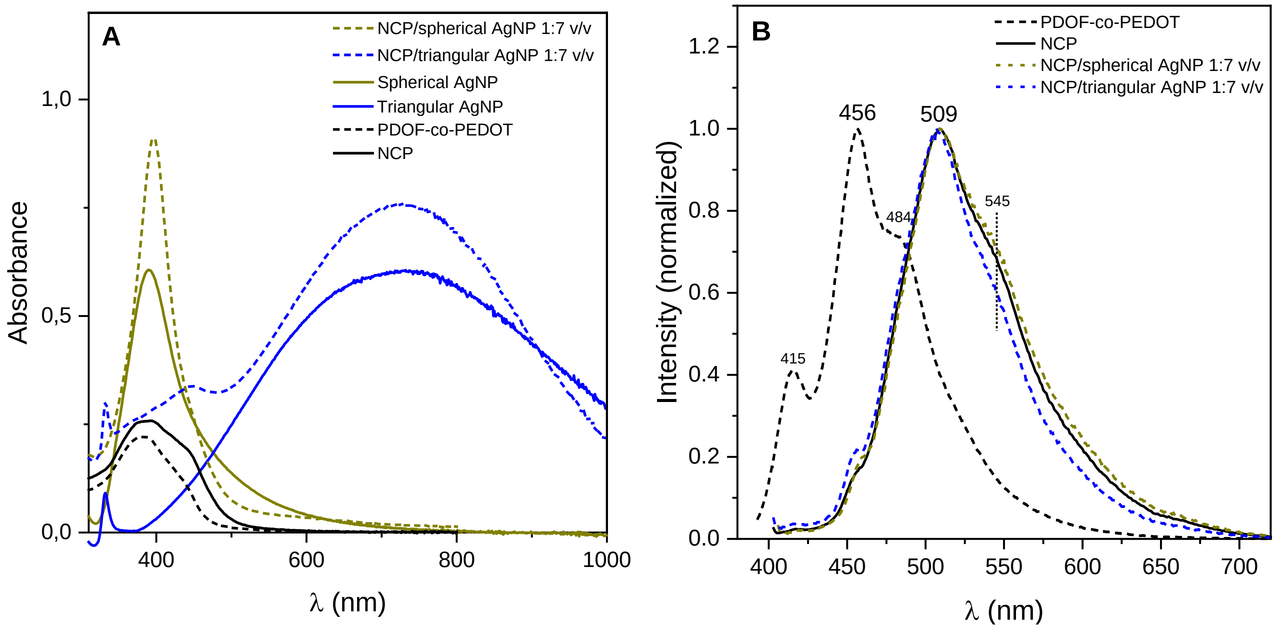

Figure S2. (**A**) Absorption spectra of the pristine PDOF-co-PEDOT (chloroform - 6 mg/L), NCP (aqueous dispersion – 60 mg/L), spherical and triangular AgNP (aqueous dispersion – 60 mg/L), and dispersions of NCP/AgNP (60 mg/L); and (**B**) Emission spectra with normalized intensity of PDOF-co-PEDOT, NCP and dispersions of NCP/AgNP.

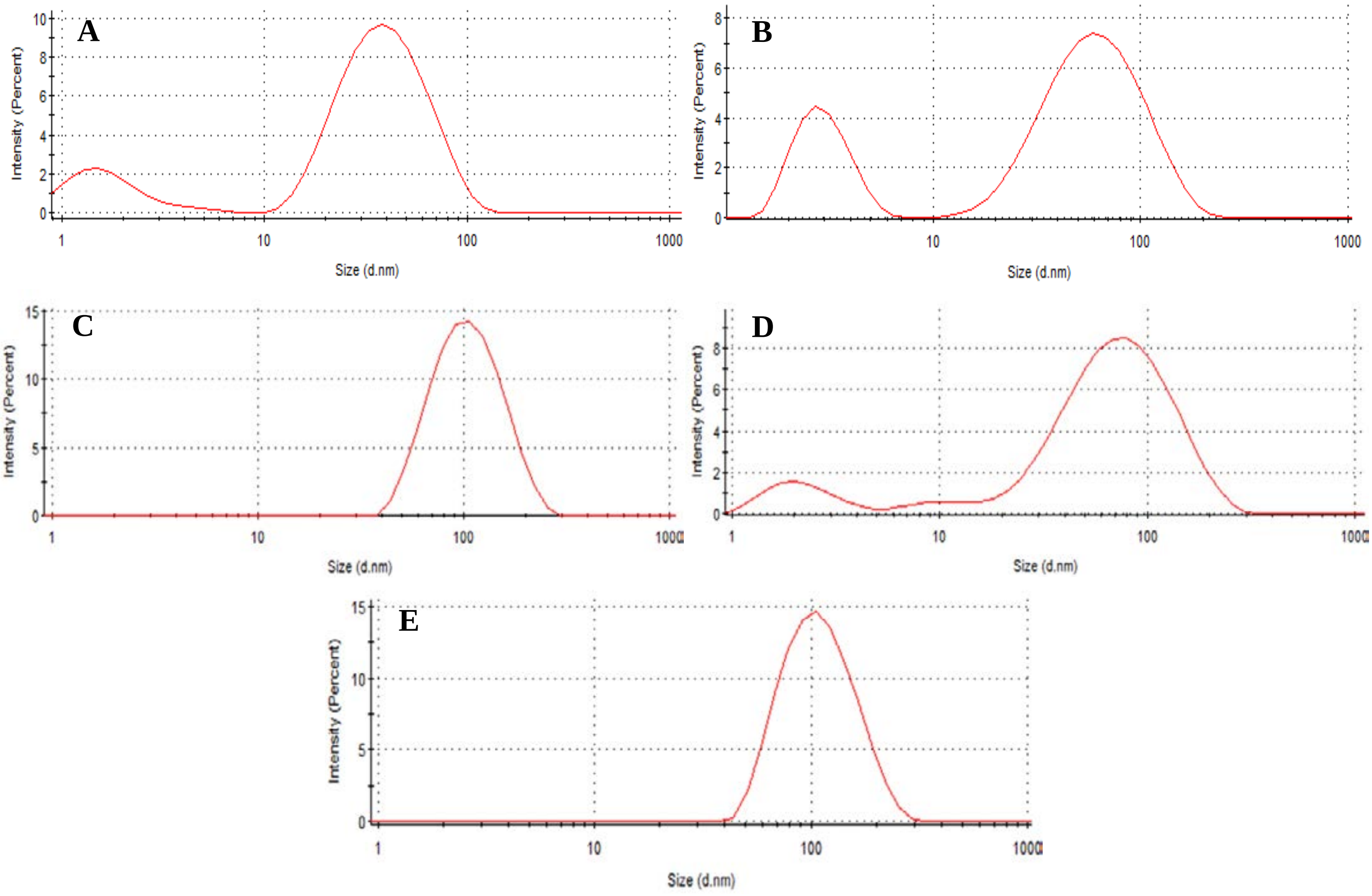

Figure S3. Particle size distribution of spherical (**A**) and triangular (**B**) AgNP, NCP/spherical AgNP (**C**), NCP/triangular AgNP (**D**) and NCP (**E**).

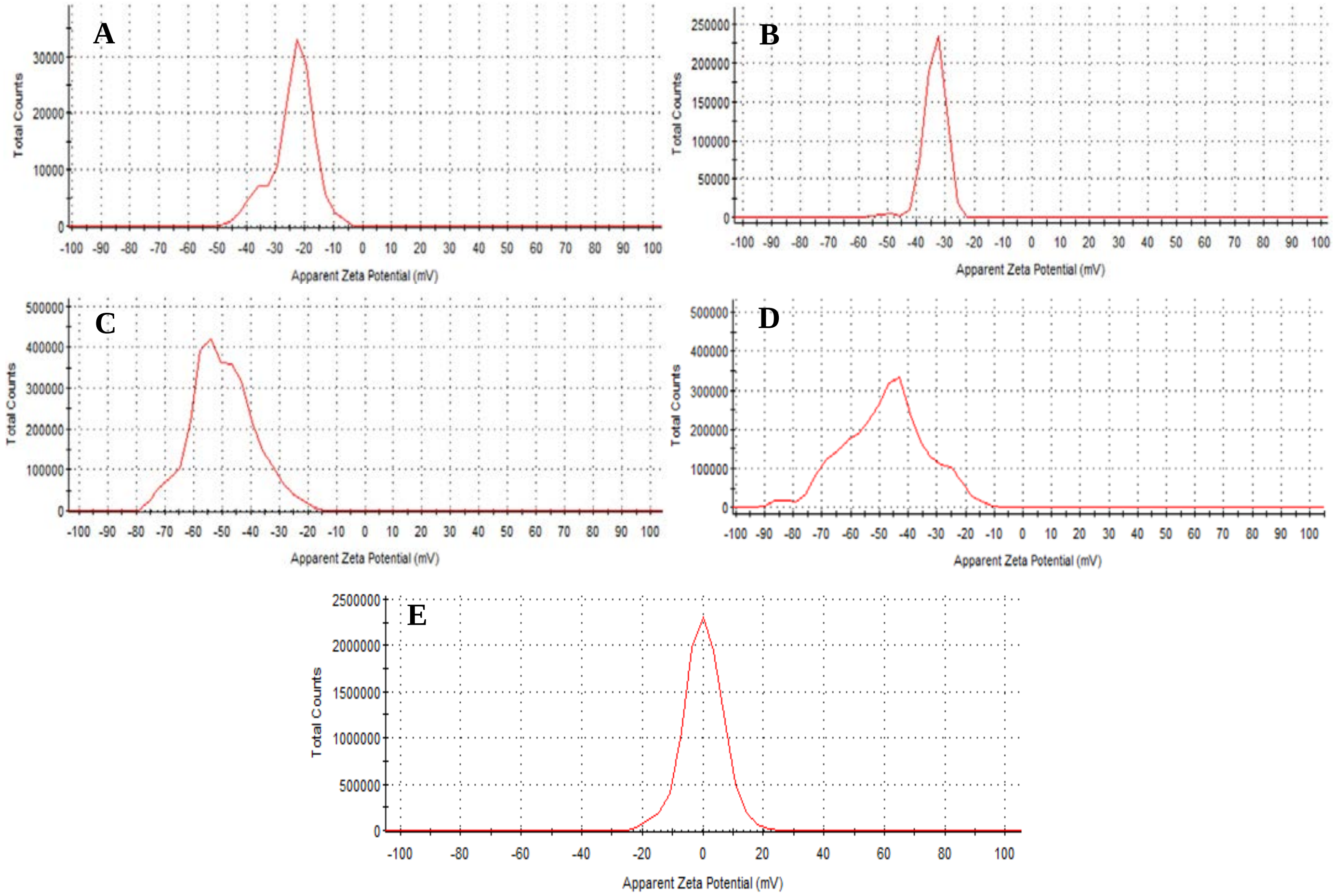

Figure S4. Zeta distribution of spherical (**A**) and triangular (**B**) AgNP, NCP/spherical AgNP (**C**), NCP/triangular AgNP (**D**) and NCP (**E**).

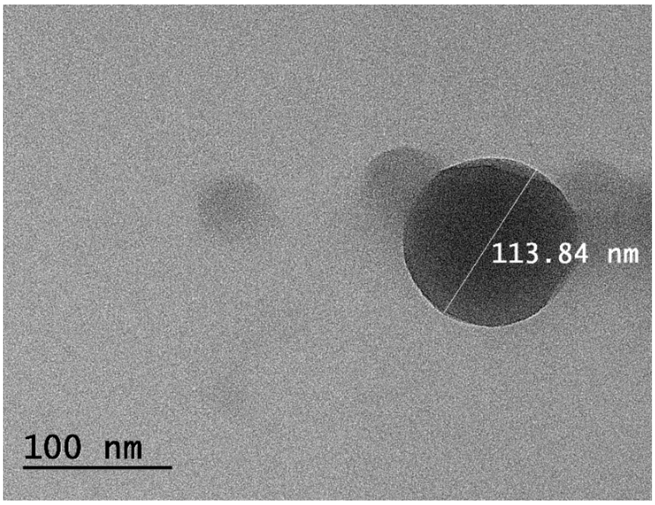

Figure S5. TEM image of NCP.

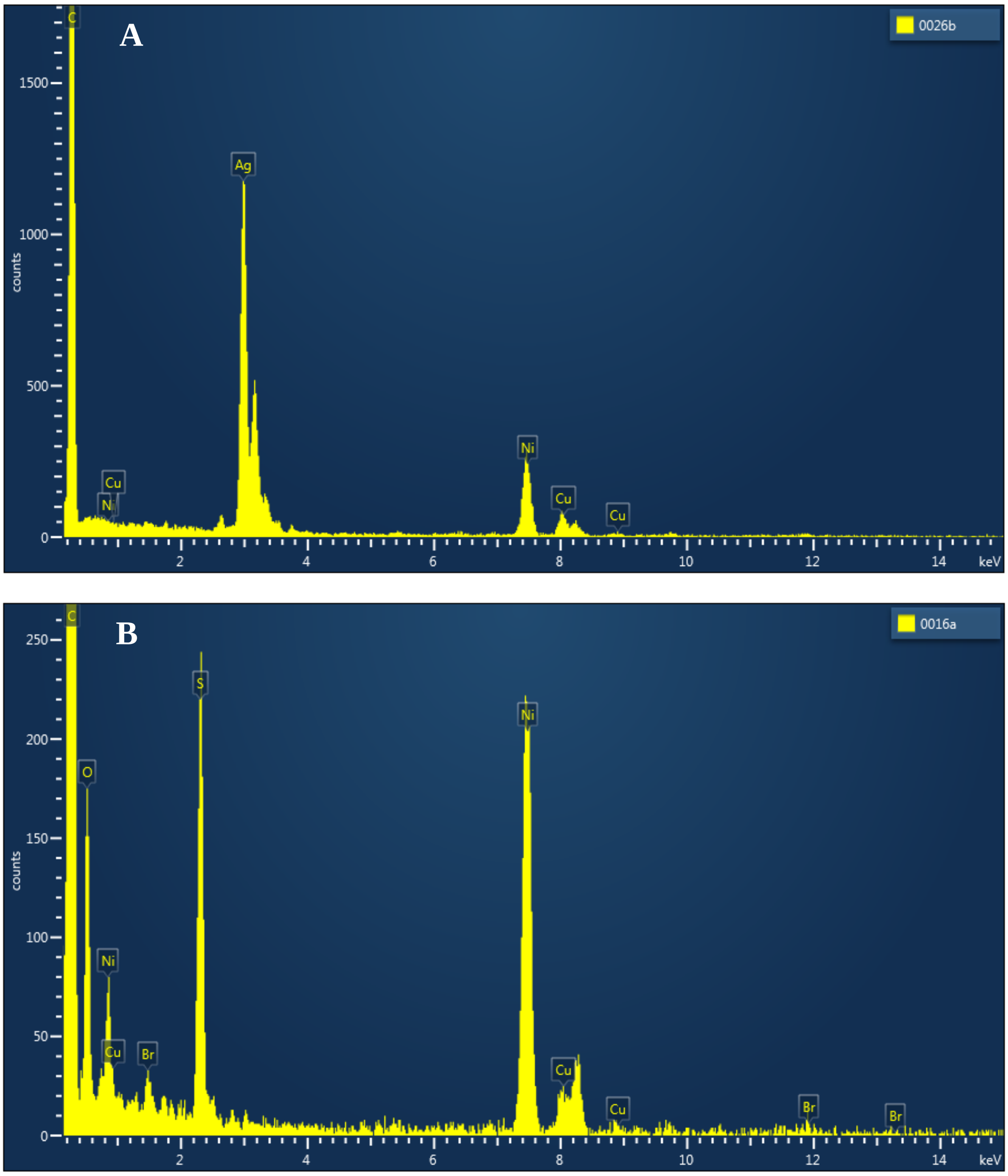

Figure S6. EDS spectra of AgNP (**A**) and NCP (**B**).

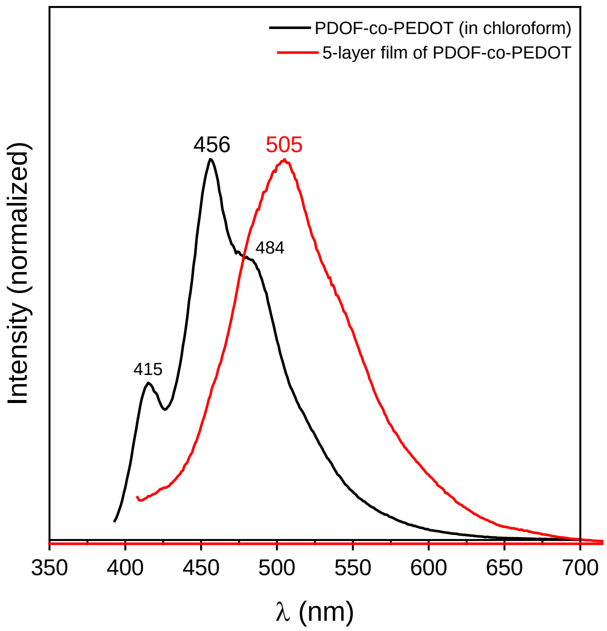

Figure S7. Emission spectra of PDOF-co-PEDOT in solution (black curve) and solid-state (red curve).

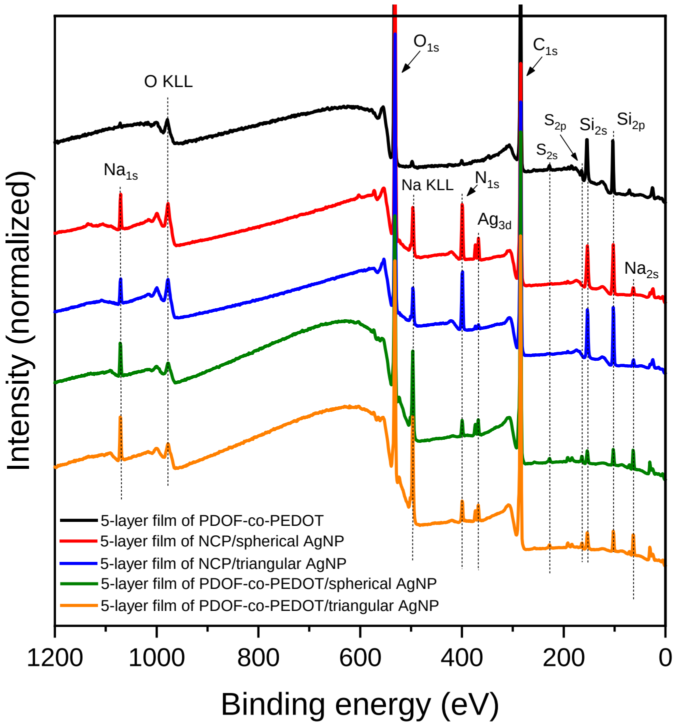

Figure S8. XPS spectra of 5-layer films of PDOF-co-PEDOT and 5-layer films of NCP or PDOF-co-PEDOT with spherical and triangular AgNP.

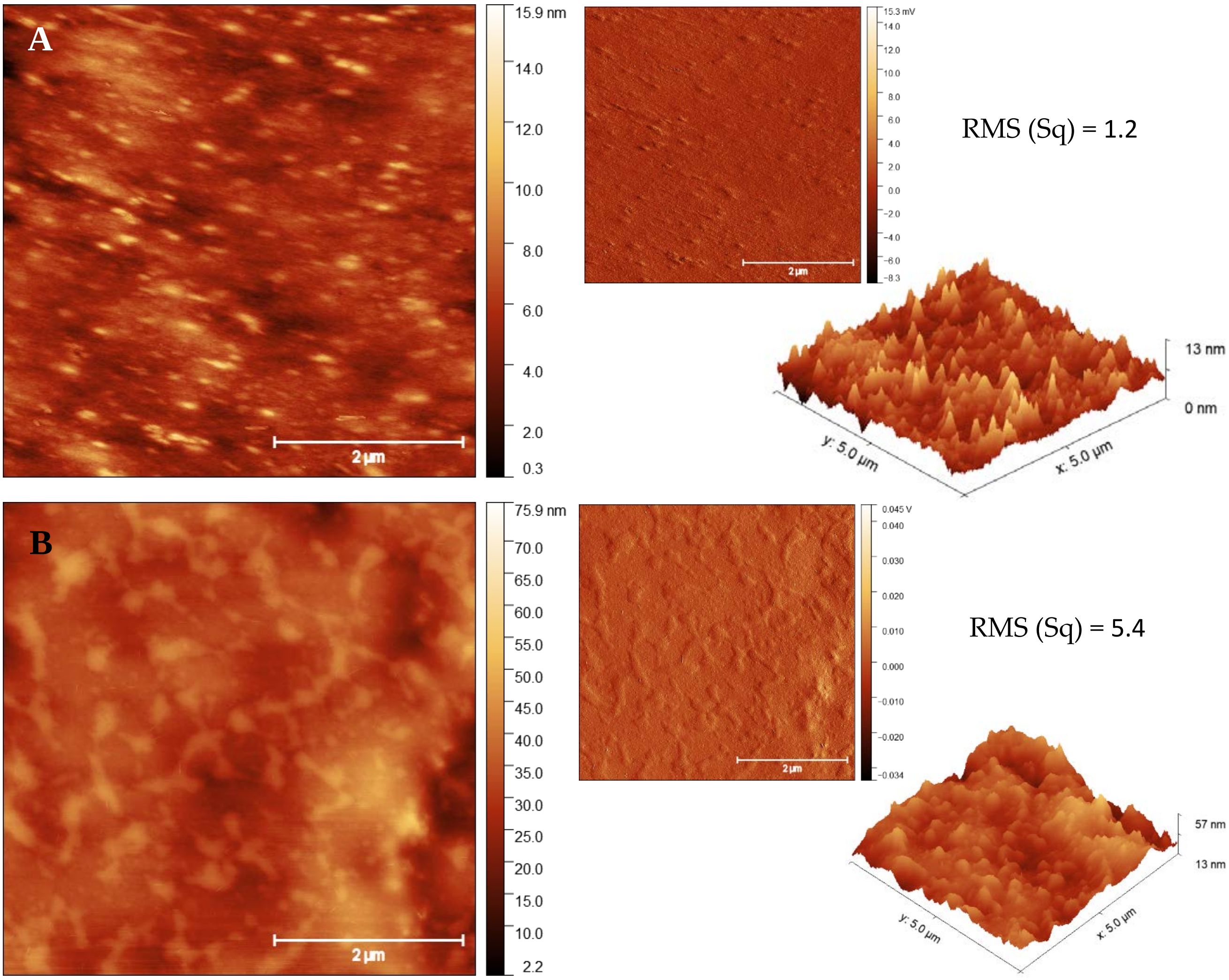

Figure S9. AFM images (5 × 5 µm) and their respective peak force error and 3D images of 5-layer films of PDOF-co-PEDOT with spherical (**A**) and triangular (**B**) AgNP.
